# Evaluation of an Indirect ELISA Using Recombinant Arginine Kinase for Serodiagnosis of *Psoroptes ovis* var. *cuniculi* Infestation in Rabbits

**DOI:** 10.3389/fvets.2019.00411

**Published:** 2019-11-20

**Authors:** Xiaobin Gu, Jiang Gu, Yongjun Ren, Youle Zheng, Guangyou Yang, Xuan Zhou, Yue Xie

**Affiliations:** ^1^Department of Parasitology, College of Veterinary Medicine, Sichuan Agricultural University, Chengdu, China; ^2^Institute of Veterinary Pharmacology, Sichuan Animal Science Academy, Chengdu, China; ^3^Animal Breeding and Genetics Key Laboratory of Sichuan Province, Sichuan Animal Science Academy, Chengdu, China; ^4^Institute of Animal Genetics and Breeding, College of Animal Science and Technology, Sichuan Agricultural University, Chengdu, China

**Keywords:** *Psoroptes ovis* var. *cuniculi*, arginine kinase, indirect ELISA, early serodiagnosis, rabbit

## Abstract

*Psoroptes ovis* var. *cuniculi* is a common ectoparasite of the wild and domestic rabbits worldwide, which causes significant economic losses in commercial rabbit breeding. In China, the diagnosis of rabbits infested with *P. ovis* var. *cuniculi* currently relies on detection of clinical signs and *Psoroptes* mites in skin scrapings by microscopy examination. However, this method is not very efficient in detection of the low mite loads and/or sub-clinical infections. In the present study, we cloned and expressed an arginine kinase homolog gene from *P. ovis* var. *cuniculi* (Poc-AK) and used its recombinant protein rPoc-AK to develop an indirect enzyme-linked immunosorbent assay (iELISA) method for diagnosis of *P. ovis* var. *cuniculi* infestation in rabbits. The results showed that the rPoc-AK protein was ~61 kDa and had no signal peptide. The rPoc-AK-based iELISA achieved a 94.4% sensitivity and a 88.2% specificity, and was able to detect *P. ovis* var. *cuniculi* infection as early as the 1st week post-infection, prior to the appearance of clinical signs. Further field study showed 24.94% (66.33/266) clinically normal rabbits were seropositive with the highest and lowest seropositive rates for California (35.71%) and Belgian (15.14%), respectively. These results suggested that the rPoc-AK has potential as a diagnostic antigen for early *P. ovis* var. *cuniculi* infestation in rabbits.

## Introduction

*Psoroptes ovis* var. *cuniculi* (also known as *Psoroptes cuniculi*) is a common ectoparasite of the wild and domestic rabbits worldwide and causes over 70% incidence rate in China (1–3). Rabbits infestations with *P. ovis* var. *cuniculi* are characterized by dermatitis, itching, hair loss, a thick crust, and a considerable negative impact on weight (2, 4–7). In China, the diagnosis of rabbits infested with *P. ovis* var. *cuniculi* currently relies on detection of clinical signs and further confirmation by observation of *Psoroptes* mites in skin scrapings using microscopy examination. This method is highly sensitive in severely affected rabbits, but low sensitive in the low mite loads and/or sub-clinical infections. Moreover, the collection and examination of skin-scrapings from rabbits are time-consuming, tedious, and impractical. Therefore, serological methods would be more convenient and efficient. The crude extracts of *Psoroptes* mites were reported to detect *P. ovis* var. *cuniculi* infestation in rabbits (8), however, the lacking *in vitro* propagation systems for *Psoroptes* mites caused the mass production problems. Thus, the recombinant antigens would be more practical for diagnosis of the psoroptic mange in rabbits. However, until now only one recombinant protein troponin C of *P. ovis* var. *cuniculi* has been evaluated and its potential as serological antigen for diagnosis of *Psoroptes* infestation was disappointing because of the extremely low specificity (9). So, there are still lacking of effective antigens for serodiagnosis of *P. ovis* var. *cuniculi* infestation in rabbits.

Arginine kinase (AK; EC 2.7.3.3), a highly conserved member of the phosphoprotokinase (PK) family, has only been observed in invertebrates (10) and is involved in energy metabolism, cell growth, immune modulation, and environmental adaptation (10–12). More importantly, the AK protein has been considered a potential diagnostic antigen for the zoonotic toxocariasis (13). In *P. ovis*, the AK has been reported to exist across all lifecycle stages of this mite (14), however, until now no other researches on *P. ovis* AK haven been reported. Herein, we cloned and expressed a novel AK homolog from *P. ovis* var. *cuniculi* (Poc-AK) and assessed its potential as a seroantigen in diagnosis of *P. ovis* var. *cuniculi* infestation in rabbits by indirect ELISA (iELISA), and then this rPoc-AK-based iELISA was further used to investigate seroprevalence of *P. ovis* var. *cuniculi* infestation in a rabbit farm.

## Materials and Methods

### Mite Source

*Psoroptes ovis* var. *cuniculi* mites were collected from a clinically infected New Zealand White rabbit (NZW), provided by the Department of Parasitology, Sichuan Agricultural University (Sichuan, China). About 300 mites, a mixture of adults, nymphs, and larvae, were harvested and stored in liquid nitrogen for subsequent RNA extraction.

### Rabbit Sera

Thirty-six *P. ovis* var. *cuniculi*-positive serum samples were collected from naturally infected rabbits at two rabbit farms located in Chengdu, Sichuan, China. *P. ovis* var. *cuniculi* infections were examined according to two gold standards, i.e., skin lesion condition and identification of *P. ovis* var. *cuniculi* mites in skin scrapings (15). Forty-seven negative serum samples from rabbits clinically and parasitologically free of *P. ovis* var. *cuniculi* were collected from rabbits raised in a farm without a history of psoroptic mange. Another 38 serum samples for cross-reactions included *Sarcoptes scabiei*-positive (*n* = 10), *Cysticercus pisiformis*-positive (*n* = 10), and *Eimeria*-positive (*n* = 18) sera, provided by the Department of Parasitology, Sichuan Agricultural University.

### Cloning, Expression, and Purification of Poc-AK

Total RNA was extracted from *P. ovis* var. *cuniculi* mites using a MiniBest universal RNA extraction kit (TaKaRa, Dalian, China) and reverse-transcribed into cDNA using a PrimeScript RT reagent Kit with gDNA Eraser (TaKaRa). According to the annotated *P. ovis* var. *cuniculi* transcriptome sequence (GenBank No. PRJNA317241) (16), the full-length sequence encoding Poc-AK was amplified from cDNA using primers 5′-CGGGATCCCCAATGCCTTCAGGTG-3′ (forward; *BamH*I restriction site underlined) and 5′-CCCTCGAGTCACATTGTTTTTTCCATT-3′ (reverse; *Xho*I restriction site underlined). The PCR product was ligated into the pET32a (+) expression vector (Invitrogen, Beijing, China), and the resulting construct was transformed into *Escherichia coli* BL21 (DE3). The recombinant protein expression was induced by 0.5 mM isopropyl-β-D-thiogalactoside (IPTG) at 37°C for 12 h. The recombinant Poc-AK (rPoc-AK) was harvested in form of the inclusion body, solubilized in 8 M urea, and purified by a Ni-NTA His-tag affinity kit (Bio-Rad, California, USA) using a step-wise elution with 20, 50, and 100 mM imidazole. The purified protein was further dialyzed against phosphate buffered saline (PBS) and concentrated using Amicon Ultra Centrifugal Filter devices (Millipore, Billerica, MA, USA) according to the manufacturer's protocol.

### Sequence Analysis

The complete gene sequence of Poc-AK was obtained and the corresponding amino acid sequence was deduced by ORF Finder (http://www.ncbi.nlm.nih.gov/gorf/orfig.cgi). DNAMAN version 7.0 was used to calculate identities between homologous genes. SignalP 4.1 (http://www.cbs.dtu.dk/services/SignalP/) was used to predict signal peptides. Transmembrane regions and subcellular localization were analyzed using the online Transmembrane Prediction Server (http://www.sbc.su.se/miklos/DAS/). The theoretical isoelectric point (pI) and molecular weight were predicted using the ExPASy server (http://web.expasy.org/protparam/).

### Recombinant Poc-AK Polyclonal Antibody Preparation

Polyclonal antibody recognizing rPoc-AK was raised as previously described (17). In brief, rabbits were immunized four times by subcutaneous injection (2 weeks apart) with 200 μg purified rPoc-AK. Sera were collected before immunization and 3 days after the final injection, then purified using HiTrap ProteinA affinity chromatography (Bio-Rad).

### Western Blotting Analysis

The purified rPoc-AK protein was separated by 12% SDS-PAGE and then transferred to a nitrocellulose membrane by a trans-blot SD semi-dry transfer cell (Bio-Rad). The membrane was blocked using 5% skim milk powder for 2 h at room temperature, then incubated with rabbit anti-*P. ovis* var. *cuniculi* serum or anti-rPC-AK IgG (1:200 v/v) overnight at 4°C. Non-infected rabbit serum was used as a negative control. After washing with TBST (0.02 M Tris-HCl, pH 7.6, 0.15 M NaCl, 0.05% Tween-20), the membrane was incubated with horseradish peroxidase (HRP)-conjugated goat anti-rabbit antibody (1:1,000 dilution; Boster Bio-project Co. Dalian, China) for 2 h at room temperature. Finally, the signal was measured using an Enhanced HRP-DAB Chromogenic Substrate Kit (Tiangen, Beijing, China).

### Establishment of an Indirect ELISA (iELISA)

An indirect ELISA was performed essentially by following Crowther and Walker's protocol (18). The checkerboard titration test was performed to ascertain the optimal concentrations of antigen and serum for the rPoc-AK-based iELISA. The 96-well plates were coated with 100 μL of two-fold dilutions of rPoc-AK protein (diluted in 0.1 M carbonate buffer pH 9.6 at 12, 6, 3, 1.5, 0.75, and 0.375 μg/well) and incubated overnight at 4°C. After three 5-min washes with phosphate-buffered saline (PBS) containing Tween-20 (PBST), plates were incubated with blocking buffer (5% skim milk diluted in PBS) for 90 min at 37°C, then with 100 μL of two-fold dilutions of *P. ovis* var. *cuniculi*-infested positive or negative sera (pooled samples from six animals; diluted in PBS at 1:20, 1:40, 1:80, 1:160, 1:320, and 1:640) for 60 min at 37°C. After washing three times, the plates were incubated with the recommended working concentration of HRP-conjugated goat anti-rabbit IgG (1:3,400; diluted in 0.01 M PBS) (Boster Bio-project Co., Wuhan, China) at 37°C for 60 min. Following five washes in PBST for 5 min each time, 100 μL of TMB chromogenic substrate solution (Tiangen) was added and the plates were placed in the dark at 37°C for 20 min. Subsequently, 100 μL of 2 M H_2_SO_4_ was added to stop the reaction, and the optical density (OD) was determined at 450 nm using a microplate reader (Thermo Scientific, Pittsburgh, PA, USA). The optimal working concentrations of antigen and sera were determined when the highest P/N value was obtained between positive and negative sera.

Under optimal conditions, 47 negative serum samples from healthy rabbits free of *P. ovis* var. *cuniculi* were used to determine the cut-off value for indirect ELISA, which serves as an identity standard for negative and positive sera. The cut-off value was calculated as the arithmetic mean OD450 value plus three standard deviations (SD) for *Psoroptes*-free rabbits (19).

### Sensitivity and Specificity of the rPoc-AK Indirect ELISA

Thirty-six serum samples from rabbits infected with *P. ovis* var. *cuniculi* were used for determination of sensitivity according to the following formula: sensitivity = (ELISA positive/true positive) × 100%. Cross-reactions were investigated with sera from rabbits infected with *S. scabiei* (*n* = 10), *C. pisiformis* (*n* = 10), and *Eimeria* (*n* = 18), respectively. In addition, 47 negative serum samples were collected from *P. ovis* var. *cuniculi*-free rabbits without a history of psoroptic mange and use to determine the specificity of the iELISA. In summary, specificity was determined using the following formula: specificity = (ELISA negative/true negative) × 100%.

### Repeatability and Reproducibility of the rPoc-AK Indirect ELISA

Five positive serum samples collected from rabbits infested with *P. ovis* var. *cuniculi* were used to evaluate repeatability and reproducibility. Repeatability (intra-assay variability) was assessed from OD450 values with triplicates in one coated ELISA plate. The same five serum samples were also applied to assess reproducibility (inter-assay variability) in another three different coated plates. These experiments were performed under the optimal conditions for the rPoc-AK indirect ELISA. The coefficients of variation (CV) for intra- and inter-assay variability were calculated according to the OD450 values and standard deviation (SD) from all samples.

### Experimental Infestation of Rabbits With *P. ovis* var. *cuniculi* and Diagnosis Testing Using the rPoc-AK Indirect ELISA

Experimental infestation of rabbits with *P. ovis* var. *cuniculi* was carried out essentially following a previously described protocol (8). In brief, 10 3-month-old naive NZW rabbits (half females and half males) were infested by placing ~200 different life-cycle stages *P. ovis* var. *cuniculi* mites deeply into the external auditory canal and closing with cotton for 1 day. All rabbit serum samples were taken weekly from week 0 until week 4 post-infection (p.i.). All 50 serum samples from rabbits experimentally infested with *P. ovis* var. *cuniculi* were then examined using the rPoc-AK indirect ELISA. Each serum sample was analyzed in triplicate, and positive and negative controls were included in all plates.

### Field Investigation of the rPoc-AK Indirect ELISA

The field investigation using the rPoc-AK indirect ELISA was carried out for analysis of 266 serum samples collected from a rabbit farm in Chengdu City, Sichuan Province, China, in October 2018. These sera were collected from clinically unaffected individual rabbits, but these subjects were from a rabbit farm with a previous history of psoroptic mange. Serum samples were collected from five breeds of rabbits (Table 1) as follows: California (*n* = 84), Hyla (*n* = 77), Belgian (*n* = 35), Qixing crossbreds (France White rabbit × Sichuan White Rabbit, *n* = 22), ZIKA × NZW crossbreed (*n* = 48). Each serum sample was tested three times.

**Table 1:** Two hundred and sixty six rabbit serum samples collected and the results of three replicates detections using rPoc-AK-based indirect ELISA.

**Variable**	**Level**	**Sample no. (Female/Male)**	**Positive sample no. (Female/Male)**			**Seropositive rate (%)**
			**Trail I**	**Trail II**	**Trail III**	
Breed	California	84 (61/23)	29 (20/9)	31 (21/10)	30 (20/10)	35.71
	Hyla	77 (51/26)	17 (12/5)	17 (12/5)	16 (11/5)	21.64
	Qi xing	22 (11/11)	4 (1/3)	6 (1/5)	5 (1/4)	22.73
	Belgian rabbit	35 (13/22)	6 (2/4)	4 (1/3)	6 (2/4)	15.14
	ZIKA × NZW	48 (26/22)	10 (6/4)	8 (3/5)	10 (3/7)	19.44
Age (months)	≥4	183	50	54	51	28.23
	<4	83	16	12	16	17.67
Gender	Female	162	41	38	37	23.87
	Male	104	25	28	30	26.61
Total		266	66	66	67	24.94

### Statistical Analysis

All data are presented as mean ± standard deviation (SD). The significance of differences between groups were tested using Mann-Whitney *U*-tests in SPSS software v.17.0 (SPSS, Inc., Chicago, Illinois, USA), and *p* < 0.05 were considered statistically significant.

## Results

### Recombinant Poc-AK and Sequence Analysis

**Figure 1:**
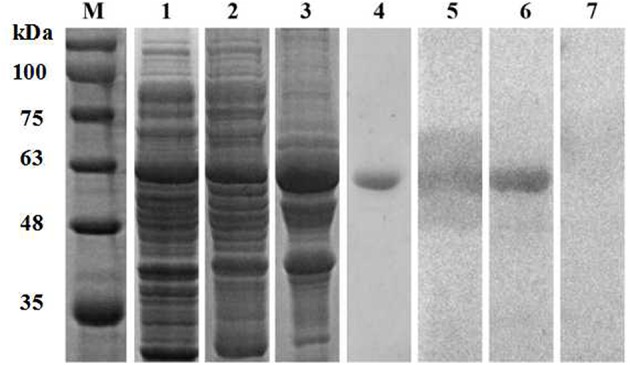
SDS-PAGE and western blotting of *P. ovis* var. *cuniculi* AK. Lanes: M, protein molecular weight markers (kDa); 1, non-purified recombinant AK; 2,3, protein solubility analysis (supernatant and inclusion bodies); 4, purified recombinant AK; 5, purified recombinant AK detected in serum from a rabbit naturally infested with *P. ovis* var. *cuniculi*; 6, purified recombinant AK detected in rabbit anti-AK serum; 7, purified recombinant AK detected in negative serum.

The full-length *P. ovis* var. *cuniculi* arginine kinase gene includes an open reading frame of 1,116 bp encoding a 371 amino acid polypeptide. The expected molecular weight of the protein is 42.9 kDa and the theoretical isoelectric point (pI) is 9.10. The Poc-AK protein has no signal peptide, no transmembrane regions, and is hydrophilic. We identified six highly conserved motifs in the deduced amino acid sequence of Poc-AK, including an arginine binding site, an ADP binding site, a substrate binding site, a synergistic substrate binding site (D78, Y84, R208, and P287) and an arginine enzyme active site residue essential for kinase activity of C286. The nucleotide sequence of Poc-AK reported in this study was identical to those reported for an arginine kinase from *P. ovis* var. *cuniculi* (PRJNA317241) (16). The amino acid alignment showed Poc-AK had identities of 52.15–82.46% with other mites including *Dermatophagoides pteronyssinus, Tetranychus urticae, Dermanyssus gallinae*, and *S. scabiei*. The full-length coding sequence of Poc-AK was successfully ligated into the pET32a (+) vector for recombinant expression of the Poc-AK in *E. coli* BL21 (DE3) cells (Figure 1, lane 1), and the fusion protein was mainly present in insoluble inclusion bodies (Figure 1, lanes 2 and 3). The purified rPoc-AK protein was of the expected molecular size (61 kDa), including an extra 20 kDa for the attached His-tag fusion peptide (Figure 1, lane 4).

### Western Blotting

Western blotting confirmed that the purified rPoc-AK ~61 kDa protein was recognized by rabbit anti-*P. ovis* var. *cuniculi* serum and anti-rPoc-AK serum IgG (Figure 1, lanes 5 and 6), which revealed this recombined Poc-AK protein with strong reactivity and antigenicity. Meanwhile, negative sera from naive rabbits did not react with the purified rPoc-AK protein (Figure 1, lane 7).

### Establishment of an Indirect ELISA (iELISA) Using rPoc-AK

In accordance with the checkerboard titration procedure, optimal conditions for iELISA were determined from the highest positive to negative ratios and then used in subsequent experiments. Optimal conditions for iELISA were 13.3 μg/mL for the coated rPoc-AK antigen per well, and a 1:320 dilution of rabbit primary serum. The cut-off value was 0.401 according to the mean OD450 value plus 3SD from 47 sera from naive rabbits using optimal conditions. Therefore, serum with OD450 ≥ 0.401 was judged positive, while OD450 <0.401 was judged negative.

**Figure 2:**
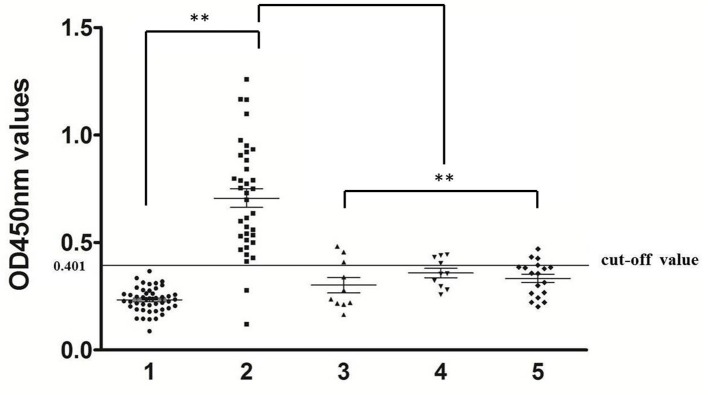
Sensitivity and specificity of indirect ELISA. 1, rabbit serum samples of uninfected *P. ovis* var. *cuniculi* (*n* = 47), 2, rabbit serum samples of infected *P. ovis* var. *cuniculi* (*n* = 36), 3, *S. scabiei* (*n* = 10), 4, *C. pisiformis* (*n* = 10), 5, *Eimeria* spp. (*n* = 18). The symbol (^**^) indicates a very significant difference (*P* < 0.01) between *P. ovis* var. *cuniculi-*positive sera and other serum samples according to the Mann-Whitney *U*-tests.

Using optimal conditions, 34/36 serum samples from rabbits naturally infected with *P. ovis* var. *cuniculi* were determined as positive (OD450 > 0.401), equating to a sensitivity of 94.4% (Figure 2, panel 2). In cross-reactivity tests, there were three *S. scabiei*-positive sera, four *C. pisiformis-*positive sera, and three *Eimeria*-positive sera giving cross-reactivity with Poc-AK, representing a specificity of 88.2% (75/85; Figure 2, panels 1, 3–5).

The coefficients of variation (CV) for intra- and inter-assay variability ranged from 0.34 to 2.13% (average = 1.13%) and from 2.13 to 6.45% (average = 4.31%), respectively. Both intra- and inter-assay CVs were below 10%, confirming that the rPoc-AK-based iELISA was stable and reproducible.

### Diagnosis Testing

**Figure 3:**
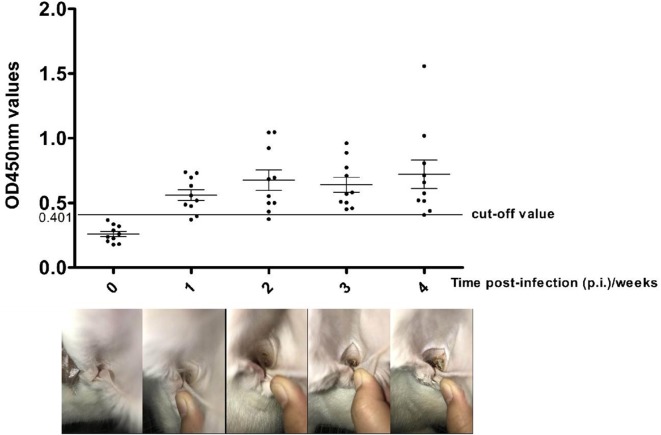
Serum antibody profiles probed by *P. ovis* arginine kinase (rPoc-AK)-based indirect ELISA in rabbits experimental infections of *P. ovis* var. *cuniculi*. The horizontal line shows the cut-off value of 0.410.

At 2 weeks p.i., a slight crust started to form at the inoculation site in all 10 experimentally-infested rabbits, and this tend increased after 3–4 weeks p.i. (Figure 3, lower lane). Changes in anti-Poc-AK antibody levels in rabbits during artificial infection with *P. ovis* var. *cuniculi* are shown in Figure 3. A higher anti-Poc-AK antibody value than cut-off value (0.401) was first detected at 1 week p.i., with 8/10 rabbit sera identified as positive (Figure 3, lane 2). Positive serum antibodies against Poc-AK gradually increased from 2 to 4 weeks p.i. (Figure 3, lanes 3–5), suggested that the rPoc-AK-based iELISA developed in this study could be used for early diagnosis of psoroptic mange in rabbits.

### Field Investigation of Seroprevalence

**Figure 4:**
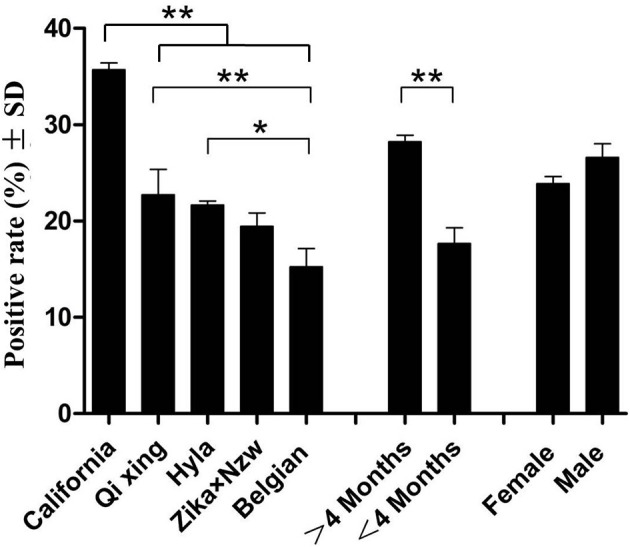
Clinical detections using rPoc-AK-based indirect ELISA. ^*^ and ^**^ indicate statistical significance at *p* < 0.05 and *p* < 0.01, respectively.

A panel of 266 serum samples from a rabbit farm were included in a field study analysis of the rPoc-AK-based iELISA (Table 1). The iELISA achieved an overall seropositive rate of 24.94% (66.33/266). Among the five rabbit breeds, the highest seropositive rate was observed for California (35.71%), followed by Qixing (22.73%), Hyla (21.64%), ZIKA × NZW crossbreed (19.44%), and Belgian (15.14%; Table 1). Moreover, the California breed yielded a significantly higher seropositive rate than that of other four rabbit breeds (*p* < 0.01), while the value for the Belgian breed was significantly lower than that of Qixing and Hyla (*p* < 0.05; Figure 4). No significant differences were observed between Qixing and Hyla (*p* > 0.05), or between Hyla and the ZIKA × NZW crossbreed (*p* > 0.05; Figure 4).

Comparison of seropositive rates of rabbits of different ages revealed that ≥4-months rabbits yielded higher seropositive rate than that of rabbits under 4 months of age (Figure 4). However, the difference in seropositive rates between sex was not statistically significant (*p* > 0.05; Figure 4).

## Discussion

To our knowledge, the arginine kinase (AK) has been reported to exist in all *P. ovis* lifecycle stages (14), however, no other research on *P. ovis* var. *cuniculi* AK has been conducted so far. In the present study, we cloned and expressed AK from *P. ovis* var. *cuniculi* and evaluated its potential application for the serodiagnosis of the mite infection in rabbits.

AK, a member of the highly conserved phosphoprotokinase (PK) family present in invertebrates, reversibly catalyzes the transphosphorylation reaction between arginine and ATP, yielding ADP and phosphagen (20). It is known to play a crucial role in cellular energy metabolism and maintaining constant ATP levels in invertebrate cells. Data on AKs in parasites are limited to a few reports on *Trypanosoma brucei* (21), *T. cruzi* (22), *Toxocara canis* (23), *Haemonchus contortus* (24), *Teladorsagia circumcincta* (25), *Ascaris suum* (26), *Lucilia cuprina*, and *Ctenocephalides felis* (27, 28). Thus, there remain large gaps in our knowledge of AK from parasitic mites. In the present study, AK from mite parasite *P. ovis* var. *cuniculi* was cloned and expressed. Comparison of deduced amino acid sequences revealed conserved regions in mite AKs and suggested all AKs possess identical substrate and arginine binding sites, i.e., a central AK region consisting of seven amino acid residues (CPTNLGT) and an essential arginine enzyme active site C276 residue (29–31).

The clinical signs of *Psoroptes* infestation in the field can be observed over a long period of time, sometimes several months (32, 33). Although microscopy examination is able to detect *P. ovis* infection with minimal clinical signs, the sensitivity is as low as 18% (15). Therefore, other approaches, especially serodiagnostic methods are urgently needed. Previous serological diagnostics based on crude extracts were reported to detect *P. ovis* infestation in sheep (34), elk (35), cattle and rabbit (8, 36). However, because of lacking *in vitro* propagation systems for *Psoroptes* mites, the recombinant proteins that can be recognized by serum antibodies raised in *P. ovis*-infested animals would be more practical for serodiagnosis of psoroptic mange. To date, the recombinant *P. ovi*s allergen, Pso o 2, is an ideal antigen in serodiagnosis of subclinical *P. ovis* infection in sheep with the high sensitivity and specificity (37–39), however, its potential serodiagnosis in detect *P. ovis* var. *cuniculi* infestation in rabbits is unknown. Zheng et al. showed that the recombinant Troponin C was not a suitable antigen candidate for detection of the *P. ovis* var. *cuniculi* infestation in rabbits because of the low specificity (25.0%) (9). In the present study, we developed a rPoc-AK-based iELISA and showed that this serological approach can achieve a 94.4% sensitivity and a 88.2% specificity. Three serum samples from *S. scabiei*-infection showed positive. Similar cross-reactions between *Psoroptes* and *Sarcoptes* infestations were also observed in other studies and have been verified as a common problem (9, 34, 40). *Psoroptes* and *Sarcoptes* mites are sometimes present in a same rabbit in nature and same acaricidal drugs are used for control of these two mite species (41, 42). In addition, seven serum samples from rabbits infection with *C. pisiformis* and *Eimeria* also showed weak cross-reactions with rPoc-AK but their OD values were significantly lower than those in the *P. ovis* var. *cuniculi* group (*p* < 0.01) and all were close to the cut-off value. These results suggested that the recombinant Poc-AK could be a potential antigen for diagnosis of the rabbit infestation of *P. ovis* var. *cuniculi* in practice.

In our study, the ELISA test showed that rPoc-AK detected anti-*P. ovis* var. *cuniculi* antibodies as early as the 1st week p.i. in the overwhelming majority of rabbits (80%) which was 1 week earlier than a slight crust emergence and also earlier than previously reported rPso o2-ELISA and crude *Psoroptes* extract-based ELISA (15, 37). The finding indicated that rPoc-AK has great potential as an antigen in diagnosing the early stages of *P. ovis* var. *cuniculi* infestation in rabbits.

In our field investigation, our rPoc-AK-based iELISA revealed a 24.94% seropositive rate in 266 clinically normal rabbits. This result indicated that our iELISA can detect asymptomatic animals during the early infestation stages of *P. ovis* var. *cuniculi*. In nature, such asymptomatic animals may be a main source of infection but are neglected when developing control strategies. Moreover, there were significant differences in seropositive rates found between different rabbit breeds in this study. The California breed yielded the highest seropositive rate (35.71%), implying its susceptibility to *Psoroptes* mites; whilst the Belgian breed displayed the lowest seropositive rate (15.14%), to certain extent indicating its resistance. However, both speculations still require further researches. In addition, seropositive rates of ≥4 months rabbit were higher in rabbits than those <4 months (28.23% vs. 17.67%, *p* < 0.01), and higher in males than females (23.87% vs. 26.61%, *p* > 0.05). Similar infection differences between age and sex were observed for elk scabies (35).

## Conclusion

Taken together, in the present study we firstly identified a homolog of arginine kinase of *P. ovis* var. *cuniculi* and developed an indirect ELISA method with high sensitivity and specificity using its recombinant protein rPoc-AK. The iELISA method can detect *P. ovis* var. *cuniculi* infestation on 1 week p.i., prior to observable disease symptoms, revealing its potential for early diagnosis of *P. ovis* var. *cuniculi* in rabbits.

## Data Availability Statement

The nucleotide sequence of arginine kinase gene from *P. ovis* var. *cuniculi* in this article is available in the GenBank database under the accession no. MN013016.

## Ethics Statement

All rabbits in this study were managed in strict accordance with the Guide for the Care and Use of Laboratory Animals (National Research Council, Bethesda, MD, USA) and the recommendations in the ARRIVE guidelines (https://www.nc3rs.org.uk/arrive-guidelines). The experiment protocol was approved by the Animal Care and Use Committee of Sichuan Agricultural University (SYXK 2014-187).

## Author Contributions

XG, JG, YR, YZ, GY, and XZ performed the experiments, analyzed the data, and drafted parts of the manuscript. XG and YX conceived and funded the study, revised, and edited the manuscript. All authors read and approved the final manuscript.

### Conflict of Interest

The authors declare that the research was conducted in the absence of any commercial or financial relationships that could be construed as a potential conflict of interest.
